# Neutrophil percentage-to-albumin ratio is an independent risk factor for MACEs in patients with acute coronary syndrome undergoing percutaneous coronary intervention

**DOI:** 10.3389/fcvm.2026.1703203

**Published:** 2026-02-05

**Authors:** Weiguo Wang, Yongxia Zhao, Kunpeng Wang, Lixian Sun, Ying Zhang, Weichao Shan

**Affiliations:** 1 Department of Cardiology, The Affiliated Hospital of Chengde Medical University, Chengde, China; 2 Hebei Key Laboratory of Panvascular Diseases, Chengde, China; 3 The Cardiovascular Research Institute of Chengde, Chengde, China

**Keywords:** acute coronary syndrome, major adverse cardiovascular events, neutrophil percentage-to-albumin, percutaneous coronary intervention, rehospitalization due to severe heart failure

## Abstract

**Purpose:**

Neutrophils are well-established biomarkers of systemic inflammation, whereas serum albumin levels are commonly used as reliable indicators of nutritional status. The neutrophil percentage-to-albumin ratio (NPAR), a novel composite biomarker integrating inflammatory and nutritional parameters, has shown promising predictive value across a range of clinical settings. However, its association with outcomes in patients with acute coronary syndrome (ACS) remains unclear. This study aimed to evaluate the prognostic utility of NPAR for predicting major adverse cardiovascular events (MACEs) in patients with ACS.

**Patients and methods:**

From January 2016 to December 2018, 1553 consecutive patients with ACS undergoing percutaneous coronary intervention (PCI) were enrolled at the Affiliated Hospital of Chengde Medical University. The NPAR was calculated as the admission neutrophil percentage divided by serum albumin concentration. Serum albumin was measured in g/L and converted to g/dL (dividing by 10) for NPAR calculation to align with conventional units. The primary follow-up endpoints were MACEs, comprising all-cause mortality and heart failure readmission.

**Results:**

During follow-up, 1,524 patients completed the study; 55 of them experienced MACEs. The NPAR levels differed significantly between the MACE and non-MACE groups (*P* < 0.001). Receiver operating characteristic (ROC) analysis demonstrated an area under the curve (AUC) of 0.700 for the NPAR in predicting MACEs (95% CI: 0.635–0.766; *P* < 0.001), with an optimal cutoff value of 17.326 determined using the Youden index. Kaplan–Meier analysis revealed significantly lower cumulative event-free survival in the high NPAR group (≥17.326) compared to the low NPAR group (<17.326) [log-rank *P* < 0.001]. Multivariable Cox regression identified four independent predictors of MACEs: age ≥ 65 years [hazard ratio [HR]: 2.944, 95% confidence interval [CI]: 1.653–5.245, *P* < 0.001]; left ventricular ejection fraction <40% (HR: 6.114, 95% CI: 2.786–13.419, *P* < 0.001); serum creatinine > 110 μmol/L (HR: 3.768, 95% CI: 1.336–10.631, *P* = 0.012), and NPAR ≥ 17.326 (HR: 3.014, 95% CI: 1.418–6.405, *P* = 0.004). Restricted cubic spline analysis confirmed a positive dose-response relationship, showing progressively increased MACE risk with rising NPAR levels (*P* < 0.001).

**Conclusion:**

NPAR ≥ 17.326 is an independent prognostic risk factor for patients with ACS undergoing PCI and may be a valuable clinical marker for identifying high-risk patients.

## Graphical Abstract

**Figure:**
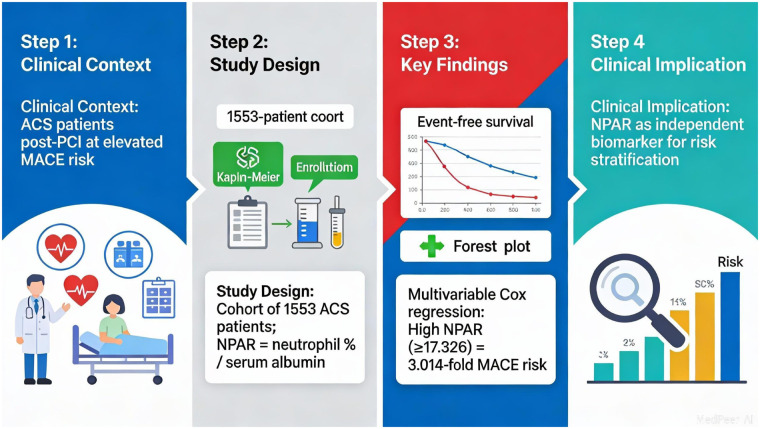
Graphical summary.

## 1 Introduction

Despite significant advancements in diagnostic techniques and therapeutic strategies, acute coronary syndrome (ACS) remains a leading cause of global mortality, imposing substantial economic and public health burdens on healthcare systems worldwide (1). Inflammation plays a pivotal role in the progression, destabilization, and rupture of atherosclerotic plaques, which are key events leading to thrombotic complications. However, the clinical utility of conventional inflammatory biomarkers for predicting prognosis in patients with ACS is often limited by insufficient specificity and suboptimal cost-effectiveness.

The neutrophil-to-albumin ratio (NPAR), a novel composite biomarker calculated by dividing the percentage of neutrophils by serum albumin concentration, captures two essential pathophysiological processes: systemic inflammation (reflected by elevated neutrophil levels) and impaired nutritional status or heightened inflammatory response (indicated by hypoalbuminemia). A growing body of evidence indicates that elevated NPAR levels are significantly associated with the presence and severity of non-alcoholic fatty liver disease [NAFLD] (2, 3), increased risk of diabetic retinopathy (DR) in individuals with diabetes mellitus (4), higher incidence of stroke-associated pneumonia, and poor functional outcomes following spontaneous intracerebral hemorrhage (5), as well as increased all-cause mortality among critically ill patients with acute kidney injury (6). Furthermore, in respiratory diseases such as chronic obstructive pulmonary disease (COPD), elevated NPAR has been identified as an independent predictor of all-cause mortality, cardiovascular mortality, and mortality due to chronic lower respiratory diseases, demonstrating superior predictive performance for 5-year all-cause mortality (7). Among critically ill patients diagnosed with coronary artery disease (CAD), elevated admission NPAR levels have been independently linked to significantly increased risks of all-cause mortality at 30, 90, and 365 days after admission (8). In addition, in patients with advanced heart failure—a severe cardiovascular condition frequently associated with ischemic heart disease—elevated NPAR has been validated as an independent prognostic marker for 1-year mortality, showing favorable discriminatory ability (AUC = 0.785) (9). Although the prognostic value of the NPAR has been well-established across various clinical settings, particularly in major cardiovascular diseases such as CAD and heart failure, its potential association with clinical outcomes in patients with ACS has not been adequately investigated. Therefore, this study aimed to evaluate the relationship between NPAR levels and the risk of major adverse cardiovascular events (MACE) in patients diagnosed with ACS who underwent percutaneous coronary intervention (PCI).

## 2 Materials and methods

### 2.1 Study population

In the present study, a total of 1,553 patients diagnosed with ACS who underwent PCI between January 2016 and December 2018 at the Department of Cardiology, the Affiliated Hospital of Chengde Medical University (Hebei, China), were consecutively enrolled. All patients underwent coronary angiography and PCI, which were performed by experienced cardiologists.

The inclusion criteria were as follows: (1) age ≥ 18 years; (2) diagnosis of clinical ACS subtypes, including ST-segment elevation myocardial infarction (STEMI), non-ST-segment elevation myocardial infarction, and unstable angina (UA), based on the 2013 ACCF/AHA Guideline for the Management of ST-Elevation Myocardial Infarction and the 2014 AHA/ACC Guideline for the Management of Patients with Non-ST-Elevation Acute Coronary Syndromes; and (3) presence of ≥50% luminal stenosis in at least one major coronary artery branch confirmed using coronary angiography.

Patients meeting any of the following criteria were excluded: (1) in-hospital death; (2) severe structural heart disease (including hypertrophic cardiomyopathy, myocarditis, or severe valvular disease); (3) active systemic inflammatory conditions such as acute/severe infections, connective tissue disorders, and secondary coronary vasculitis; (4) end-stage organ dysfunction, including severe hepatic impairment (Child–Pugh C) and end-stage renal disease (eGFR < 15 mL/min/1.73 m²); (5) prior coronary artery bypass grafting; and (6) critical baseline data missing (>10% variables).

This study was approved by the Ethics Committee of the Affiliated Hospital of Chengde Medical University (approval Number: CYFYLL2021036) and conducted in accordance with the ethical principles stated in the Declaration of Helsinki. All participants provided written informed consent prior to enrollment.

### 2.2 Baseline demographics and clinical characteristics

The cardiovascular research team collected comprehensive data on the demographic and clinical characteristics of the enrolled patients. Hypertension was defined as a resting systolic blood pressure ≥ 140 mmHg and/or diastolic blood pressure ≥ 90 mmHg or a prior physician diagnosis of hypertension with current antihypertensive therapy (10). Type 2 diabetes mellitus was defined as either: (1) the presence of classic diabetes symptoms (e.g., polyuria, polydipsia, unexplained weight loss) plus a random plasma glucose level ≥ 11.1 mmol/L; (2) a fasting plasma glucose level ≥ 7.0 mmol/L; (3) a 2 h plasma glucose level ≥ 11.1 mmol/L during a 75 g oral glucose tolerance test; or (4) in the absence of classic symptoms, the confirmation of hyperglycemia meeting any of the above criteria (fasting, random, or 2 h OGTT) on at least two separate occasions (11). Dyslipidemia was defined as a serum total cholesterol level ≥ 5.18 mmol/L, high-density lipoprotein cholesterol level ≤ 1.04 mmol/L, low-density lipoprotein cholesterol (LDL-C) level ≥ 3.37 mmol/L, or triglyceride level ≥ 1.7 mmol/L or a prior physician diagnosis of dyslipidemia with current lipid-lowering medication (12).

### 2.3 Follow-up and endpoints

Follow-ups were conducted by cardiovascular physicians using a standardized protocol to minimize bias. Data were collected during clinical visits at 1, 3, 6, and 12 months, and annually thereafter. The primary endpoint was major adverse cardiovascular events (MACE), defined as a composite of all-cause mortality and heart failure-related rehospitalization.

### 2.4 Statistical analysis

The Kolmogorov–Smirnov test was used to assess the normality of continuous variables. Variables that followed a normal distribution were presented as mean ± standard deviation, whereas those with skewed distributions were summarized using the median and interquartile range. Differences in non-normally distributed continuous variables between the MACEs and non-MACEs groups were analyzed using the Mann–Whitney U test. Categorical variables were expressed as frequencies and percentages and compared using the chi-square (*χ*²) test. The Kaplan–Meier method was used to estimate the cumulative incidence of adverse cardiovascular events across groups, and the log-rank test was applied to assess statistically significant differences between survival curves. Receiver operating characteristic (ROC) analysis was performed to evaluate the diagnostic accuracy of NPAR, with the optimal cutoff value determined by maximizing the Youden index (sensitivity + specificity − 1). To assess the robustness of the optimal cut-off value of NPAR determined by the Youden index, we conducted Bootstrap internal validation. Using random sampling with replacement, 1,000 Bootstrap samples (each *n* = 1,524) were generated from the final analysis cohort. In each sample, the ROC analysis was rerun to recalculate the optimal NPAR cut-off value for predicting MACE. Time-dependent ROC curves were further constructed to assess the predictive performance of the NPAR across different time points. A potential dose-response relationship between the NPAR and the risk of MACEs among patients with ACS undergoing PCI was explored using restricted cubic spline (RCS) regression. Univariate and multivariate Cox proportional hazards models were conducted to determine the prognostic value of the NPAR in predicting the risk of MACEs.

All statistical analyses were performed using SPSS (version 27.0.1, SPSS Inc., Chicago, IL, USA), GraphPad Prism 8.0 (GraphPad Software Inc., La Jolla, CA, USA), and R version 4.3.3. A two-sided *P* value < 0.05 was considered statistically significant, and no correction was made for multiple comparisons.

### 2.5 Data imputation for missing values

The rate of missing baseline variables in this study was below 5% for all included variables. To maximize data utilization and minimize potential bias, we performed data imputation as follows: for missing values in continuous variables, imputation was performed with the median value within clinically relevant subgroups (e.g., ACS subtypes); for missing values in categorical variables, imputation was performed with the mode of that variable. All subsequent statistical analyses were conducted based on the imputed dataset.

## 3 Results

### 3.1 Patients' characteristics

**Figure 1:**
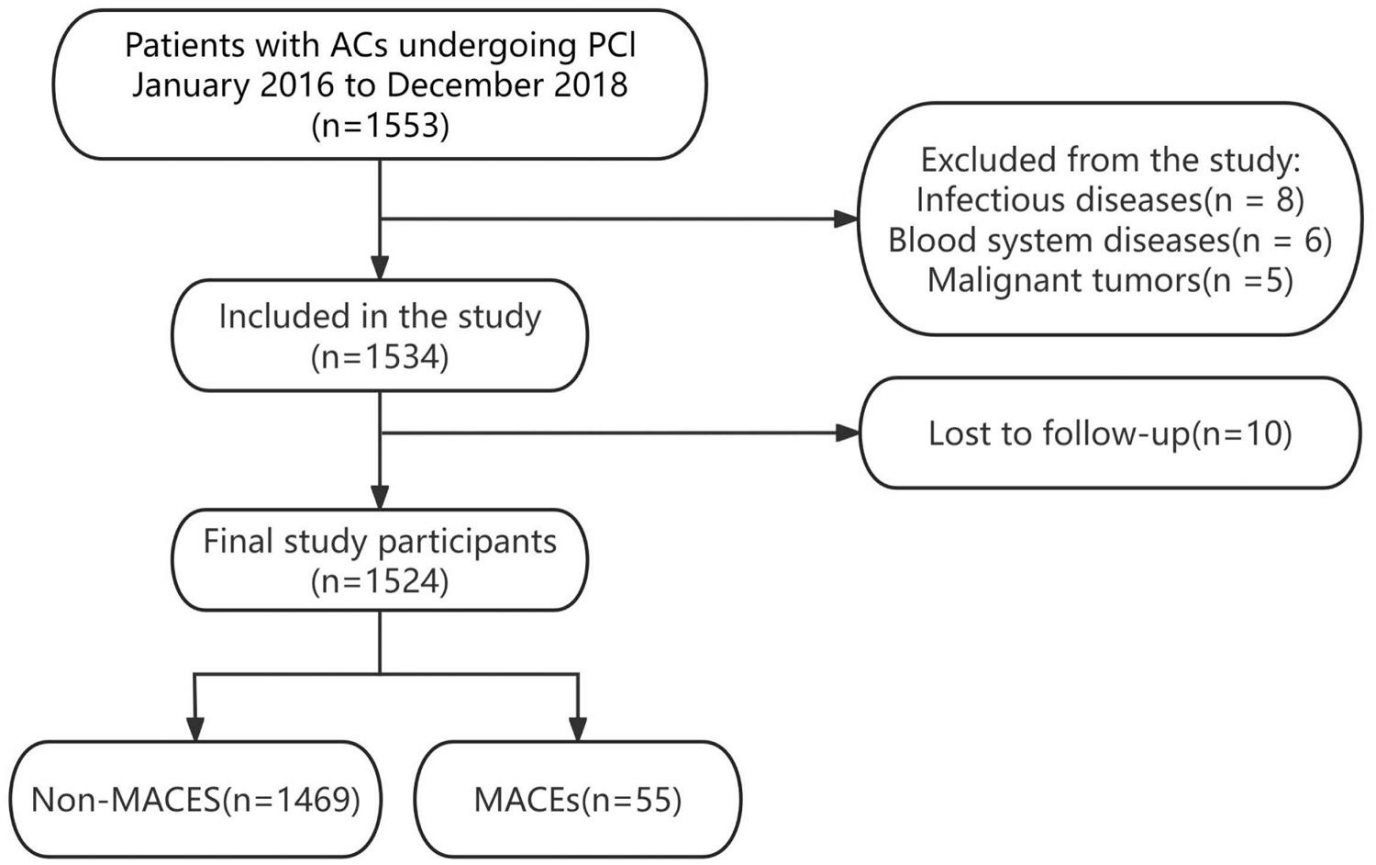
Study flowchart.

Of the 1,553 patients with ACS, 29 were excluded from the analysis. Exclusions were due to concomitant infectious diseases (*n* = 8), coexisting hematologic disorders (*n* = 6), a diagnosis of acute myocardial infarction with malignant tumors (*n* = 5), or loss to follow-up (*n* = 10). Ultimately, 1,524 patients who completed the follow-up were included, with a median follow-up duration of 1,123 days (approximately 3.0 years) (Figure 1).

Among the 1,524 patients with ACS who underwent PCI, 55 experienced MACEs, including 51 all-cause deaths and four rehospitalizations attributable to heart failure. Table 1 presents the baseline clinical characteristics stratified by MACE occurrence. Significant intergroup differences (*P* < 0.05) were observed in age, serum creatinine (sCr), left ventricular ejection fraction (LVEF), ischemic stroke, creatine kinase MB (CK-MB), unstable angina (UA), and NPAR values between the MACEs and non-MACEs groups.

**Table 1:** Baseline Patient characteristics of the MACEs and non-MACEs groups.

Variables	MACEs group (*n* = 55)	Non-MACEs group (*n* = 1,469)	*χ*2/Z	*P*-value
Demographic				
Male	40 (72.7%)	1,102 (75.0%)	0.148	0.700
Age (years)	65.30 ± 8.11	58.67 ± 10.32	−5.097	<0.001
Dyslipidemia	34 (61.8%)	833 (56.7%)	0.565	0.452
Hypertension	31 (56.4%)	865 (58.9%)	0.139	0.709
Diabetes mellitus	14 (25.5%)	375 (25.5%)	0.001	0.990
Ischemic stroke	14 (25.5%)	204 (13.9%)	5.787	0.016
Family history of CAD	4 (7.3%)	212 (14.4%)	2.234	0.135
Laboratory data				
WBC (10^9^/L)	8.89 ± 3.35	8.82 ± 3.34	−1.018	0.209
HGB (g/L)	141.09 ± 13.79	146.83 ± 15.06	−1.505	0.593
PLT (10^9^/L)	208.62 ± 55.06	219.99 ± 55.59	−0.356	0.722
NEUT (10^9^/L)	6.36 (4.86,8.96)	4.99 (3.69,7.42)	−3.274	0.001
TC (mmol/L)	4.42 ± 1.19	4.45 ± 1.05	−0.330	0.742
TG (mmol/L)	1.28 (0.79,2.20)	1.60 (1.05,2.44)	−2.003	0.052
HDL-C (mmol/L)	1.12 ± 0.28	1.12 ± 0.31	−0.601	0.548
LDL-C (mmol/L)	2.46 ± 0.94	2.41 ± 0.85	−0.014	0.989
CK-MB (U/L)	40.00 (11.00,123.49)	16.27 (10.00,48.96)	−2.325	0.020
sCr (umol/L)	81.91 ± 29.25	69.21 ± 16.47	−2.820	0.005
UA (umol/L)	334.08 ± 87.97	329.50 ± 92.21	−0.418	0.676
TP (g/L)	69.95 (64.93,78.38)	71.30 (66.70,75.45)	−0.132	0.895
ALB (g/L)	41.67 ± 4.62	40.75 ± 3.75	−3.309	0.001
NPAR	26.423 ± 37.73	16.562 ± 5.38	−1.937	<0.001
LVEF	52.55 ± 12.20	57.21 ± 8.69	−2.590	0.010
LVEDD	51.30 ± 6.59	50.90 ± 4.97	−0.088	0.930
Clinical classification of ACS				
UA	14 (25.5%)	595 (40.5%)	5.004	0.025
STEMI	29 (52.7%)	646 (44.0%)	1.646	0.200
Non-STEMI	12 (21.8%)	228 (15.5%)	1.585	0.208
Coronary angiography				
One-vessel lesion	13 (23.6%)	461 (31.4%)	1.484	0.223
Two-vessel lesion	19 (34.5%)	469 (31.9%)	0.167	0.683
Three-vessel lesion	23 (41.8%)	539 (36.7%)	0.599	0.439

Data are presented as *n* (%) or median (range).

Data are expressed as count (percentage) for categorical variables, mean ± standard deviation or median (interquartile range) for numerical variables.

CAD, coronary artery disease; WBC, white blood cell; HGB, hemoglobin; PLT, platelet; NEUT, neutrophils; TC, total cholesterol; TG, triglyceride; HDL-C, high-density lipoprotein cholesterol; LDL-C, low density lipoprotein cholesterol; CK-MB, creatine kinase MB; Cr, creatinine; UA, uric acid; TP, total protein; ALB, albumin; NPAR, neutrophil percentage-to-albumin ratio; LVEF, left ventricular ejection fraction; UA, unstable angina; STEMI, ST-segment elevation myocardial infarction; Non-STEMI, non-ST-segment elevation myocardial infarction.

### 3.2 ROC curve, survival analysis, and time-dependent ROC

**Figure 2:**
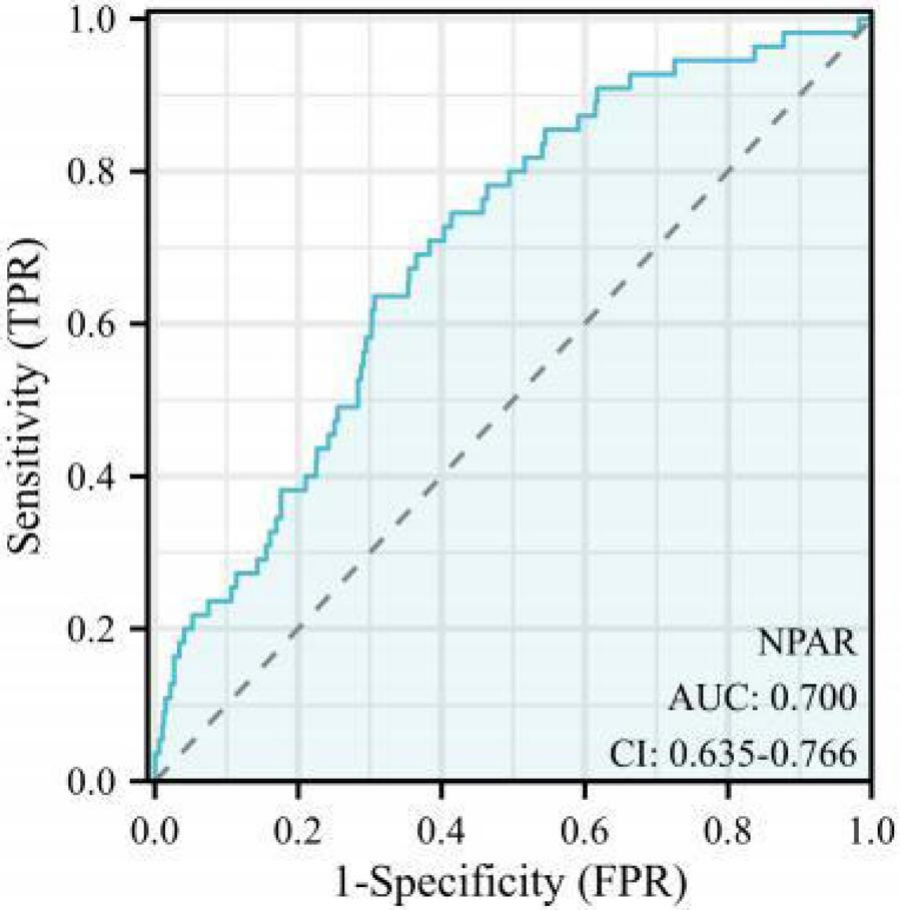
Receiver operating characteristic (ROC) curve. NPAR, neutrophil percentage-to-albumin.

The ROC curve was constructed to evaluate the discriminatory ability of the NPAR in predicting MACEs. The area under the curve (AUC) for the NPAR was 0.700 [*P* < 0.001, 95% confidence interval (CI): 0.635–0.766] (Figure 2). According to the Youden index, the optimal diagnostic cut-off value for the NPAR was 17.326, yielding a sensitivity of 57.853% and a specificity of 82.143%. Accordingly, patients with ACS were categorized into the low NPAR (<17.326) and high NPAR (≥17.326) groups based on this threshold.

### 3.3 Bootstrap internal validation

**Figure 3:**
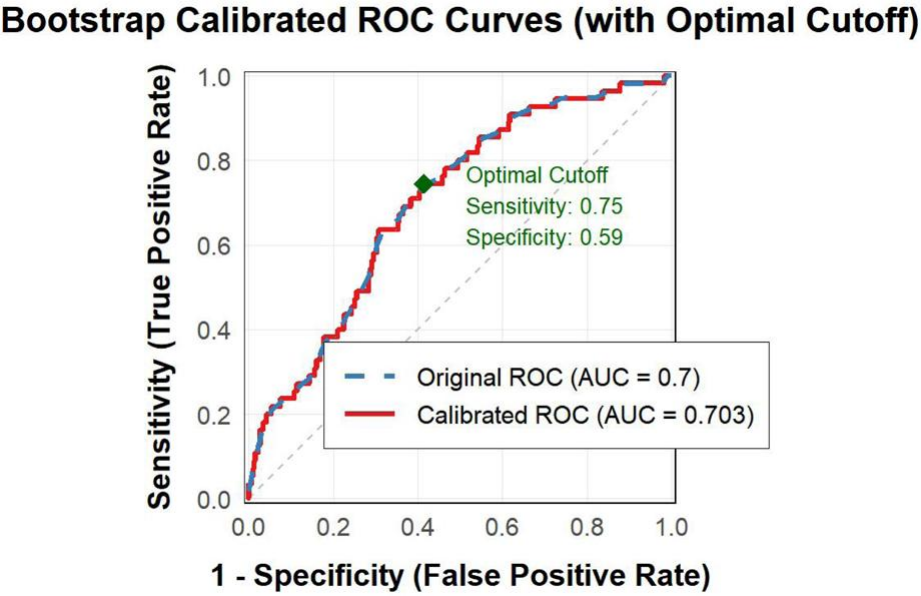
Bootstrap calibrated ROC curves.

Bootstrap resampling analysis (1,000 iterations) was performed to validate the stability of the NPAR-based prediction model. The calibrated ROC curve showed an AUC of 0.703, which was highly consistent with the original AUC of 0.700 (Figure 3). At the optimal cut-off value (17.326), the sensitivity and specificity were 0.75 and 0.59, respectively, further confirming the robustness of the NPAR as a predictive marker within this cohort.

**Figure 4:**
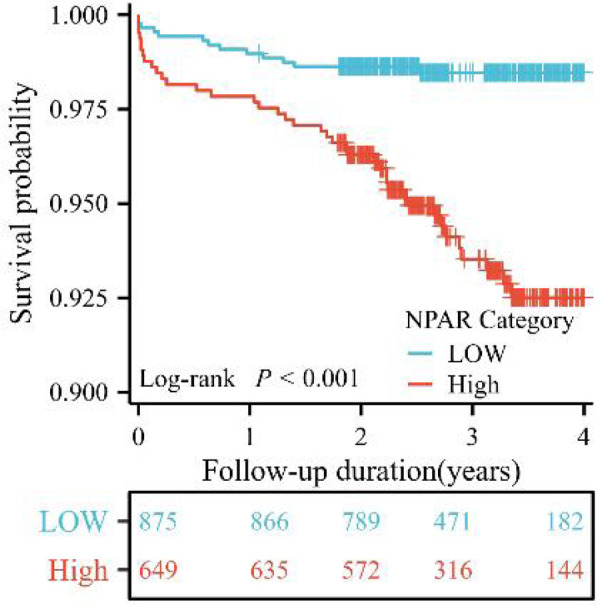
Kaplan–Meier curves of cumulative survival by NPAR in patients with ACS undergoing PCI (log-rank *P* < 0.001).

The Kaplan–Meier curve (Figure 4) demonstrated that the high NPAR group exhibited significantly lower cumulative survival rates compared to the low NPAR group (log-rank *P* < 0.001).

**Figure 5:**
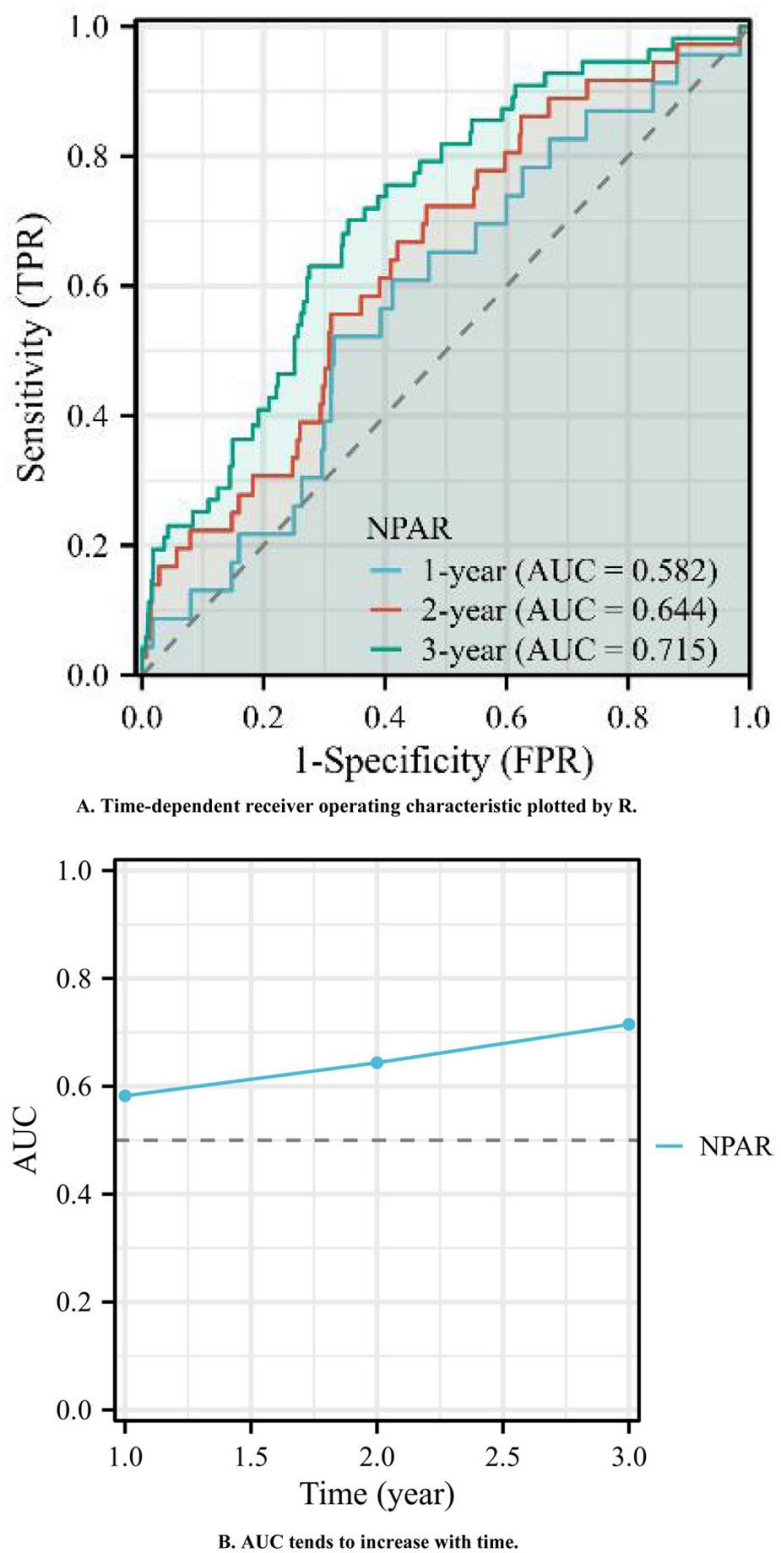
**(A)** Time-dependent receiver operating characteristic plotted by R. **(B)** AUC tends to increase with time.

A time-dependent ROC analysis was subsequently performed to assess the temporal evolution of predictive performance. Figure 5A illustrates the time-dependent ROC curves, with 1-, 2-, and 3-year AUC values of 0.582, 0.644, and 0.715, respectively. As shown in Figure 5B, the time-dependent AUC analysis revealed that the NPAR maintained consistent predictive performance for the incidence of MACEs across various time intervals. Collectively, these results suggest that the NPAR demonstrates strong and sustained predictive utility for MACEs.

### 3.4 Univariate and multivariate Cox hazard proportional models

As shown in Table 1, age, white blood cell count, ischemic stroke, sCr, CK-MB level, NPAR, LVEF, and UA showed significant differences. We transformed the continuous variables into categorical variables according to the following criteria: age ≥ 65 years old; white blood cell count ≥ 10 × 10^9^/L, CK-MB ≥ 32 U/L, LVEF < 40%, and sCr > 110 μmol/L. Thereafter, we repeated univariate COX analysis. The results still showed significant differences (all *P* < 0.05) (Table 2).

**Table 2:** Univariate Cox hazard proportional model for predictive factors of MACEs.

Variables	Univariate HR (95% CI)	*P*-value
Age ≥ 65 years	3.323 (1.957–5.642)	<0.001
Ischemic stroke	2.089 (1.139–3.832)	0.017
sCr > 110 μmol/L	2.989 (1.080–8.273)	0.035
CK-MB > 32 U/L	2.463 (1.416–4.282)	0.001
LVEF < 40%	8.182 (3.831–17.473)	<0.001
UA	0.490 (0.267–0.899)	0.021
NPAR ≥ 17.326	4.051 (2.208–7.432)	<0.001

**Figure 6:**
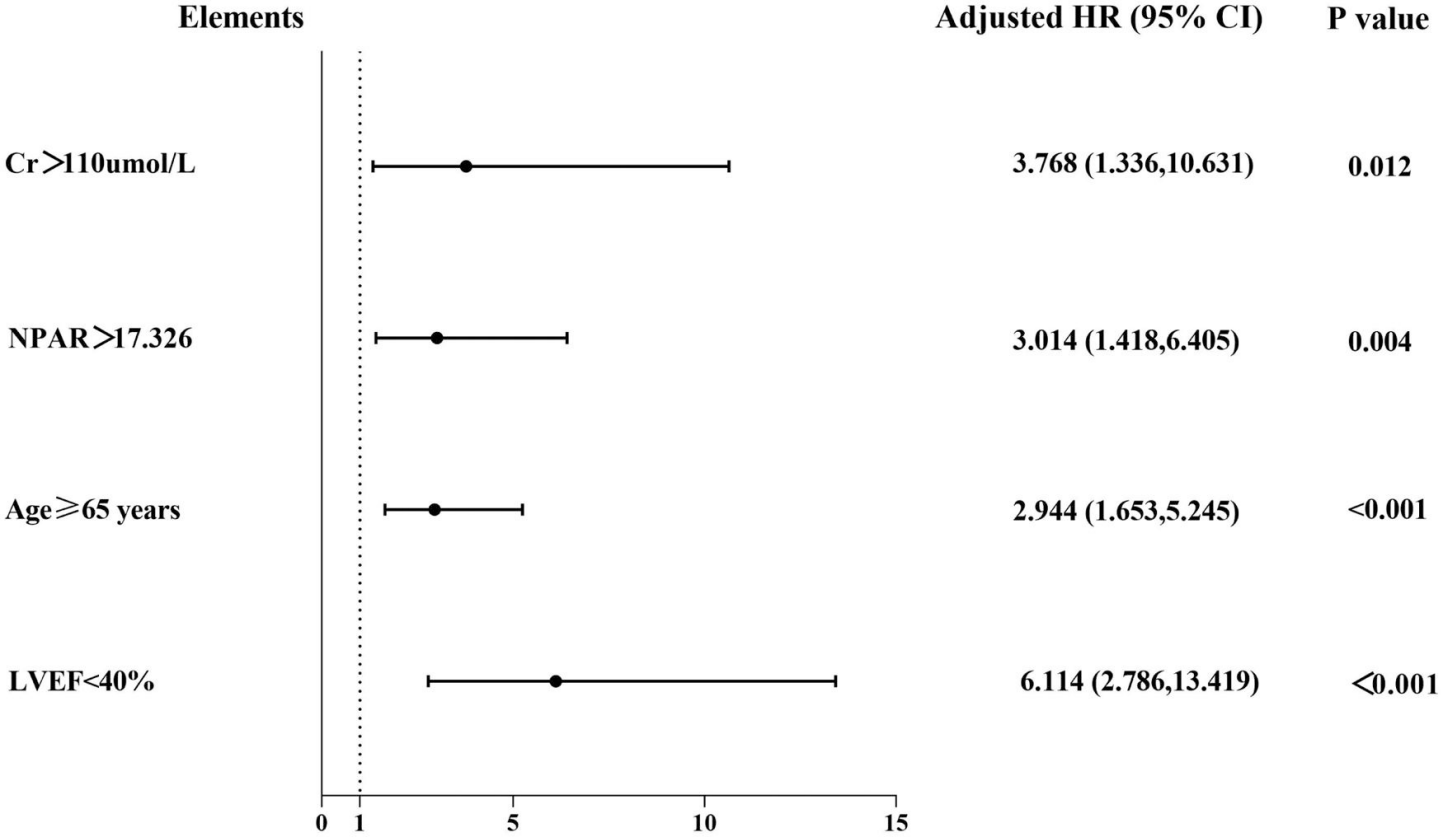
Forest graphs according to Cox proportional hazards regression model to test the risk factors for MACEs.

Additionally, factors demonstrating clinical prognostic significance in univariate Cox proportional hazards analysis—age ≥ 65 years, LVEF < 40%, sCr > 110 μmol/L, and NPAR ≥ 17.326—were included in the multivariate Cox proportional hazards model. The results revealed the following independent predictors of MACEs: age ≥ 65 years (HR: 2.944, 95% CI: 1.653–5.245, *P* < 0.001); LVEF < 40% (HR: 6.114, 95% CI: 2.786–13.419, *P* < 0.001); sCr > 110 μmol/L (HR: 3.768, 95% CI: 1.336–10.631, *P* = 0.012); and NPAR ≥ 17.326 (HR: 3.014, 95% CI: 1.418–6.405, *P* = 0.004) (Table 3, Figure 6). Therefore, an elevated NPAR (≥17.326) independently correlated with an increased MACE risk in patients with ACS.

**Table 3:** Multivariate Cox hazard proportional model for predictive factors of MACEs.

Variables	Multivariate HR (95% CI)	*P*-value
sCr > 110 μmol/L	3.768 (1.336–10.631)	0.012
LVEF < 40%	6.114 (2.786–13.419)	<0.001
Age ≥ 65 years	2.944 (1.653–5.245)	<0.001
NPAR ≥ 17.326	3.014 (1.418–6.405)	0.004

MACEs, major adverse cardiovascular events; LVEF, left ventricular ejection fraction; NPAR, neutrophil percentage-to-albumin ratio; sCr, serum creatinine; UA, unstable angina; CK-MB, creatine kinase MB.

### 3.5 RCS

**Figure 7:**
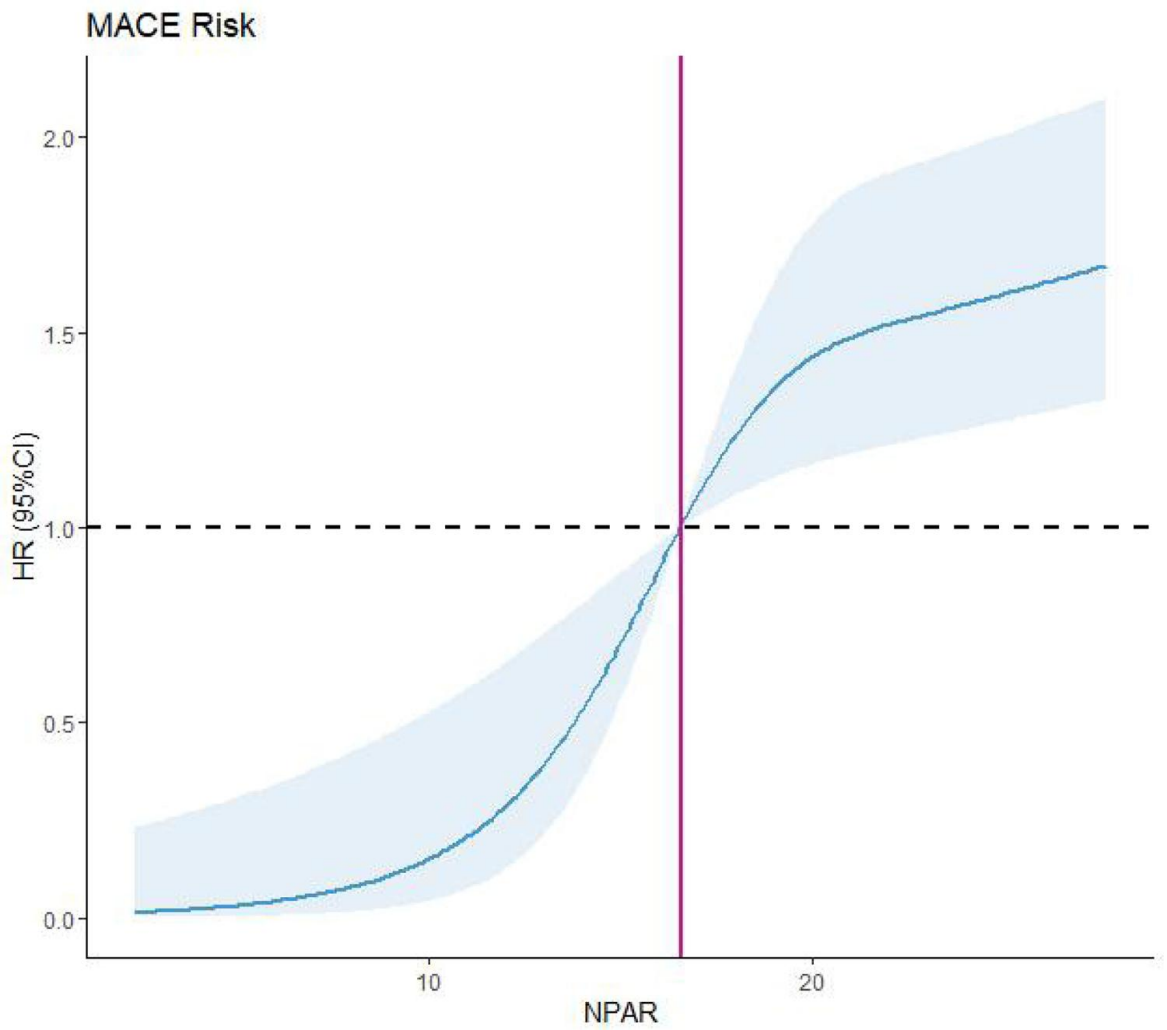
Restricted cubic spline (RCS). MACEs, major adverse cardiovascular events; NPAR, neutrophil percentage-to-albumin.

We further assessed the nonlinear relationship between the NPAR and MACEs using RCS. Elevated NPAR levels were significantly associated with increased MACE risk (*P* < 0.001 and *P*_nonlinear_ = 0.0056; Figure 7). The RCS curve demonstrated a monotonic increase in MACE risk with rising NPAR values. Collectively, these findings establish elevated NPAR as an independent risk factor for MACEs in PCI-treated patients with ACS.

## 4 Discussion

In the present study, we investigated the prognostic value of the NPAR in patients with ACS undergoing PCI. The key findings are as follows: (1) elevated NPAR was independently associated with adverse clinical outcomes and served as an independent risk factor for poor prognosis in this patient population; (2) the prognostic performance of the NPAR demonstrated relative stability and sustained predictive value over time; (3) the NPAR demonstrated significant predictive power for all-cause mortality following PCI in patients with ACS; and (4) higher NPAR levels were significantly correlated with older age, elevated sCr levels, and reduced LVEF. To our knowledge, this is the first study to comprehensively evaluate the association between this novel biomarker ratio (NPAR) and long-term prognosis in ACS patients undergoing PCI.

Accumulating evidence underscores the pivotal role of chronic, low-grade inflammation in the initiation, progression, and destabilization of atherosclerotic plaques, culminating in acute events such as myocardial infarction (13, 14). This process is characterized by complex interactions among immune cells, cytokines, and vascular components, which promote endothelial dysfunction, plaque rupture, and thrombosis (14). Consequently, inflammatory biomarkers have emerged as crucial indicators for risk stratification and prognosis, complementing traditional factors such as LDL-C (13–16). While LDL-C deposition within the arterial intima represents a primary initiating event, the subsequent oxidation of LDL particles triggers a robust inflammatory response (13). This recruits circulating leukocytes—particularly monocytes and neutrophils—to the vessel wall, driving the inflammatory cascade and contributing to plaque vulnerability (13, 14, 16). This understanding solidified inflammation as a bona fide therapeutic target in secondary prevention. Among the various inflammatory cells implicated in cardiovascular disease, neutrophils—the most abundant leukocyte subset—have garnered significant attention due to their strong predictive value for adverse outcomes, particularly mortality (15–17). Studies consistently demonstrate that the neutrophil-to-lymphocyte ratio (NLR)—a readily available and inexpensive marker reflecting systemic inflammation and stress—serves as a powerful independent predictor of poor outcomes in patients with ST-elevation myocardial infarction (STEMI) undergoing primary PCI. An elevated NLR upon admission is strongly associated with impaired coronary blood flow after PCI (no-reflow phenomenon) (15, 17) and significantly higher rates of in-hospital MACEs, including mortality (15, 17). Mechanistically, the prognostic importance of neutrophils likely stems from their direct involvement in vascular injury. Growing evidence firmly supports a central role for neutrophils and related biomarkers as key mediators of inflammation-driven cardiovascular injury and mortality.

Serum albumin, synthesized exclusively by the liver, is a critical multifunctional protein whose circulating levels reflect a complex interplay between nutritional status and systemic inflammation (18). Hypoalbuminemia frequently indicates an underlying inflammatory state, characterized by elevated pro-inflammatory cytokines such as IL-6 and TNF-alpha. These cytokines suppress hepatic albumin synthesis and increase capillary permeability, thereby reducing circulating albumin levels (18). In patients with cancer, decreased serum albumin levels are significantly associated with an increased risk of venous thromboembolism and overall mortality, independent of kidney or liver function and other inflammatory markers (19). Similarly, in patients with inflammatory bowel disease (IBD), the serum albumin-to-globulin ratio is significantly lower compared to that in non-IBD controls and demonstrates a negative correlation with established disease activity indices (20). In the context of cardiovascular diseases, hypoalbuminemia is associated with impaired patient quality of life and reduced overall longevity. In patients with acutely decompensated heart failure, hypoalbuminemia is prevalent and independently associated with a significantly increased risk of 1-year mortality (21). Furthermore, among patients hospitalized with ACS, serum albumin levels ≤ 3.50 g/dL at admission are strongly and independently predictive of the development of new-onset heart failure and in-hospital mortality (22). This body of research highlights the potential clinical utility of composite biomarkers that integrate albumin with inflammation indices.

The NPAR has emerged as an accessible biomarker integrating inflammatory and nutritional pathways. Growing evidence supports its prognostic utility across cardiovascular conditions. In atrial fibrillation, elevated NPAR independently predicted all-cause mortality, reflecting its capacity to capture systemic inflammation implicated in arrhythmia progression (23). Similarly, among patients with chronic heart failure, NPAR > 3.5 conferred a 2.1-fold increased mortality risk after multivariable adjustment, outperforming conventional inflammatory markers such as CRP (24). Although the NPAR demonstrates an established prognostic value in acute coronary events including STEMI (25), its utility in post-PCI ACS populations remains underexplored. Beyond cardiovascular contexts, NPAR elevation correlates with advanced liver fibrosis in NAFLD (2), asthma severity (26), depression (27), malignancy (28), and chronic kidney disease (28). This cross-disease validity indicates that NPAR reflects fundamental pathophysiological processes such as neutrophilic inflammation compounded by hypoalbuminemia-driven endothelial dysfunction and oxidative stress (24, 29). Collectively, the NPAR represents a cost-effective prognostic biomarker, with persistently elevated levels consistently associated with adverse outcomes across cardio-metabolic conditions.

This finding is consistent with our results. We used several methods to investigate the correlation between the NPAR and prognosis. The results showed that the NPAR may be a useful clinical indicator for predicting the risk of MACE in patients with ACS after PCI. Multivariate Cox proportional hazards model analysis revealed that age, sCr > 110 μmol/L, LVEF < 40%, and high NPAR were the main prognostic factors. It is noteworthy that our study did not include other established inflammatory biomarkers such as C-reactive protein (CRP) or the neutrophil-to-lymphocyte ratio (NLR) for direct comparison, as these were not routinely collected in our retrospective cohort. This limitation notwithstanding, NPAR retains a distinct practical advantage: it is derived exclusively from a complete blood count and serum albumin measurement—tests that are routinely performed, inexpensive, and universally available at admission for ACS patients. Thus, NPAR offers an immediately applicable tool for early risk stratification without necessitating additional specialized assays. Future prospective studies incorporating a broader panel of inflammatory markers are needed to definitively establish the incremental or comparative prognostic value of NPAR.

Age significantly modifies the clinical presentation, management, and outcomes of ACS. Large multinational registries demonstrate that advancing age fundamentally alters clinical phenotypes: each decade of life decreases the likelihood of STEMI presentation by 18%, with older patients predominantly manifesting non-ST-elevation ACS (30). This shift has important clinical implications, as older patients often exhibit atypical symptoms, complex coronary anatomy, and increased vulnerability to complications (31). Beyond chronological age, the concept of “vascular age,” which quantifies biological arterial aging, offers more accurate cardiovascular risk stratification than chronological age alone (32). Importantly, age remains an independent predictor of adverse events. Consistent evidence shows that older patients with ACS experience higher rates of in-hospital mortality, heart failure, and bleeding complications compared to younger patients, even after adjusting for comorbidities and treatment strategies (31). A better understanding of these mechanisms may help identify strategies to reduce age-related risks and improve long-term outcomes following PCI.

LVEF represents a critical biomarker for stratifying the severity of left ventricular dysfunction in patients with ACS following PCI, significantly affecting therapeutic decisions and prognostic predictions. Current guidelines underscore the necessity of LVEF assessment in ACS management (33), recognizing its pivotal role in risk stratification and guiding interventions aimed at improving survival and preventing adverse cardiovascular events. Large-scale registry data confirm a strong inverse relationship between admission LVEF and mortality risk. Patients with preserved LVEF (≥50%) exhibit significantly lower short-term and 1-year mortality rates compared to those with mild-moderate dysfunction (LVEF 30%–49%), who show better outcomes than those with severe LVEF impairment (<30% or <40%) (34, 35). Consequently, LVEF serves as a fundamental parameter for identifying high-risk patients with ACS who may benefit from more intensive monitoring and aggressive secondary prevention strategies (34, 36). Furthermore, although risk factors associated with low LVEF at presentation are increasingly recognized (34, 35), the specific risk factors contributing to mortality within distinct LVEF categories differ. This underscores the need for tailored risk assessment and management strategies based not only on the LVEF value but also on the specific clinical context and accompanying factors. Therefore, LVEF remains an indispensable biomarker in ACS, providing critical information on the degree of myocardial damage, stratifying short and long-term mortality risk, and guiding therapeutic decisions. Addressing these gaps is essential to enhance risk prediction accuracy and optimize the management of patients with ACS, ultimately improving outcomes in this high-risk population, especially given the critical observation that each 1% reduction in LVEF significantly increases the risk of in-hospital death.

PCI plays a pivotal role in the management of ACS, significantly reducing mortality rates and the recurrence of ischemic events. However, this procedure is associated with inherent risks, including contrast-induced acute kidney injury, a well-documented complication linked to the administration of iodinated contrast media during coronary angiography and interventional procedures (37). sCr, a commonly used and easily accessible biomarker of renal function, serves as an essential parameter for risk stratification in patients undergoing PCI. Extensive observational cohort studies consistently demonstrate a linear association between elevated baseline sCr levels and increased all-cause mortality among patients receiving PCI. Importantly, this association remains statistically significant even after comprehensive adjustment for potential confounding factors and across multiple sensitivity analyses (38). Moreover, studies examining early post-procedural renal changes have shown that even minor subclinical increases in sCr within 24 h after coronary angiography or PCI—often deemed clinically irrelevant—are independently related to a markedly increased risk of long-term mortality (39). Collectively, these findings highlight that sCr, both as a baseline indicator and marker of acute renal alterations, offers substantial prognostic value that surpasses traditional cardiovascular risk factors.

This study confirmed the clinical utility of the NPAR as a novel, comprehensive, and clinically applicable biomarker for assessing the long-term risk of MACEs in patients with ACS who undergo PCI. Consistent with findings reported by Han et al. (40), the NPAR is calculated from routinely available and cost-effective hematological parameters, providing meaningful insights for early risk stratification and improved clinical decision-making. This facilitates the development of more effective therapeutic strategies aimed at preventing adverse cardiovascular outcomes while advancing precision medicine without imposing additional financial burden on healthcare systems. For patients with elevated NPAR levels, intensified monitoring protocols can be implemented, including increased follow-up frequency, proactive management of modifiable risk factors, and the incorporation of advanced imaging techniques. Furthermore, individualized secondary prevention measures—targeted nutritional support, albumin supplementation in cases of hypoproteinemia, initiation of anti-inflammatory therapies, and systematic screening for latent infections—can be integrated into clinical practice. Although the NPAR demonstrates promising potential for guiding risk stratification and individualized interventions, further research is required to fully establish its clinical validity and applicability.

Collectively, these findings underscore the prognostic value of the NPAR in identifying cardiovascular risk among patients with ACS who have undergone PCI.

## 5 Study limitations

The neutrophil percentage-to-albumin ratio (NPAR) is a straightforward composite index reflecting aspects of both malnutrition and systemic inflammation. Its primary strengths lie in its simplicity, objectivity, and immediate availability from routine admission blood tests, which are universally performed in the management of ACS. Despite these advantages, our study has several limitations. First, this was a single-center study with a limited sample size; therefore, the findings may not be fully generalizable to broader populations with different demographic characteristics, access to healthcare resources, or treatment practices. Second, the retrospective design introduces potential selection bias and relies on the accuracy and completeness of data extracted from electronic medical records. Unmeasured or inadequately measured confounders may also contribute to residual bias. Third, although the median follow-up of 3 years is sufficient to assess intermediate-term risk, the stability of NPAR's predictive performance over longer periods and its long-term prognostic value require further investigation. Fourth, the number of observed endpoint events was relatively low (*n* = 55), with the composite outcome predominantly driven by all-cause mortality (51 events). Although the events-per-variable ratio in our final multivariate model was acceptable, the limited event count increases the risk of overfitting and necessitates cautious interpretation of the estimates. External validation in larger cohorts is essential. Fifth, as a retrospective analysis, our study did not systematically collect other established inflammatory biomarkers—such as high-sensitivity C-reactive protein (hs-CRP) or the neutrophil-to-lymphocyte ratio (NLR)—which precludes a head-to-head comparison of their prognostic utility against NPAR. Although this limits our ability to contextualize NPAR within the existing biomarker landscape, it also underscores NPAR's inherent practicality: it is calculated from routine admission laboratory parameters that are universally available, inexpensive, and require no additional testing. Future prospective studies should include a standardized panel of inflammatory and nutritional biomarkers to rigorously evaluate the independent and additive prognostic value of NPAR. Sixth, although internal validation via bootstrap resampling indicated stability of the NPAR cut-off value within this dataset, external validation in independent, multi-center cohorts remains necessary. Finally, the exact mechanistic association between elevated NPAR values and the occurrence of MACEs warrants further exploration.

## 6 Conclusion

Elevated NPAR was independently associated with increased risks of all-cause mortality and heart failure-related readmission in patients with ACS following PCI. Therefore, the NPAR may serve as a clinically useful biomarker for identifying PCI-treated patients with ACS at a heightened risk of MACEs.

## Data Availability

The original contributions presented in the study are included in the article/Supplementary Material, further inquiries can be directed to the corresponding author.
